# Accelerated Scheme to Predict Ring-Opening Polymerization Enthalpy: Simulation-Experimental Data Fusion and Multitask Machine Learning

**DOI:** 10.1021/acs.jpca.3c05870

**Published:** 2023-12-06

**Authors:** Aubrey Toland, Huan Tran, Lihua Chen, Yinghao Li, Chao Zhang, Will Gutekunst, Rampi Ramprasad

**Affiliations:** † School of Materials Science & Engineering, Georgia Institute of Technology, Atlanta, Georgia 30332, United States; ‡ School of Computational Science and Engineering, Georgia Institute of Technology, Atlanta, Georgia 30332, United States; ¶ School of Chemistry and Biochemistry, Georgia Institute of Technology, Atlanta, Georgia 30332, United States

## Abstract

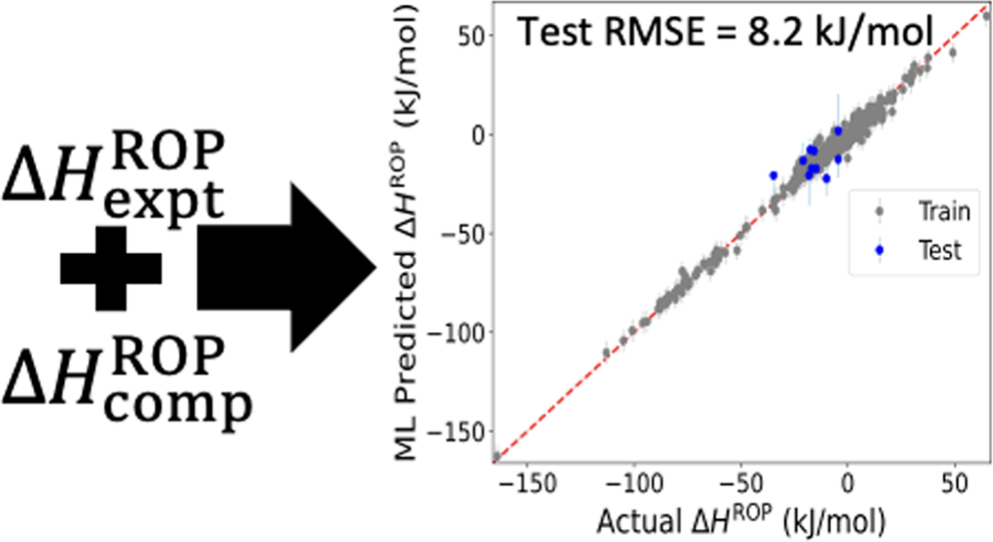

Ring-opening enthalpy (Δ*H*^ROP^) is a fundamental thermodynamic quantity controlling the polymerization and depolymerization of an important class of recyclable polymers, namely, those created from ring-opening polymerization (ROP). Highly accurate first-principles-based computational methods to compute Δ*H*^ROP^ are computationally too demanding to efficiently guide the design of depolymerizable polymers. In this work, we develop a generalizable machine-learning model that was trained on experimental measurements and reliably computed simulation results of Δ*H*^ROP^ (the latter provides a pathway to systematically increase the chemical diversity of the data). Predictions of Δ*H*^ROP^ using this machine-learning model require essentially no time while the prediction accuracy is about ∼8 kJ/mol, approaching the well-known chemical accuracy. We hope that this effort will contribute to the future development of new depolymerizable polymers.

## 1 Introduction

The superior stability, adaptability, and cost-effectiveness of polymers have led them to widespread use,^1,2^ but, on the other hand, have also created an enormous challenge for modern human civilization.^3−8^ As of 2021, only 5% of about 51 million tons of plastic created in the United States was successfully recycled,^7^ leaving the remaining material for landfilling as the main method of “storing” polymer/plastic waste. The difficulty of polymer recycling is largely due to their inherent thermodynamic, thermal, chemical, and mechanical stability. However, this hurdle has motivated a great deal of recent research activities in designing and developing recyclable polymers.^9−12^

Chemical recycling, in which polymer waste is depolymerized back to monomers before purifying and repolymerizing them, is a preferable approach.^13−16^ A main advantage of chemical recycling (compared to mechanical recycling) is that polymers produced from the recovered monomer feedstocks can preserve their purity and all of their original properties. Among numerous families of polymers, those created by opening cyclic monomers and polymerizing them are, in principle, depolymerizable and thus being particularly suitable for chemical recycling.^10−12,15,17^ This affinity for chemical recycling seen for polymers polymerized via ring-opening polymerization (ROP) is owed to the preferable thermodynamics these polymerizations tend to have.^15^ Furthermore, the polymerizability/depolymerizability equilibrium of such polymers may be adjusted by controllable parameters, such as ring-elemental chemistry, side group functionalization, and the monomer ring size. Therefore, research and development activities aiming at understanding, engineering, and designing (depolymerizable) polymers via ROP have been very active in the context of sustainability.^10,12,15,17−19^

Perhaps the most important readily tunable ROP quantity is the enthalpy of polymerization (Δ*H*^ROP^), defined as the difference between the internal energies of the resulting polymers and the monomers used in the polymerization process. This thermodynamic quantity, which is closely related to the monomer ring size and the ring strain, can be measured^19,20^ and computed^18,21−25^ at reasonable levels of fidelity. Traditionally, Δ*H*^ROP^ was computed by opening a ring monomer atomic configuration (believed to be its ground state), passivating the dangling bonds by suitable end groups, and then computing the energies using first-principles computations.^21−25^ This procedure is simple, but reaching acceptable accuracy is challenging.^18^ The main reason could be traced back to the soft-material nature of polymers, which are certainly not locked into any single atomic configuration, especially at and above room temperatures. Therefore, another method has recently been developed^18^ that adequately samples the space of polymer and monomer atomic configurations at the level of first-principles computations for better estimation of Δ*H*^ROP^. While this advanced method is significantly more robust and accurate than the traditional method, it is also very computationally demanding.^18^

**Figure 1:**
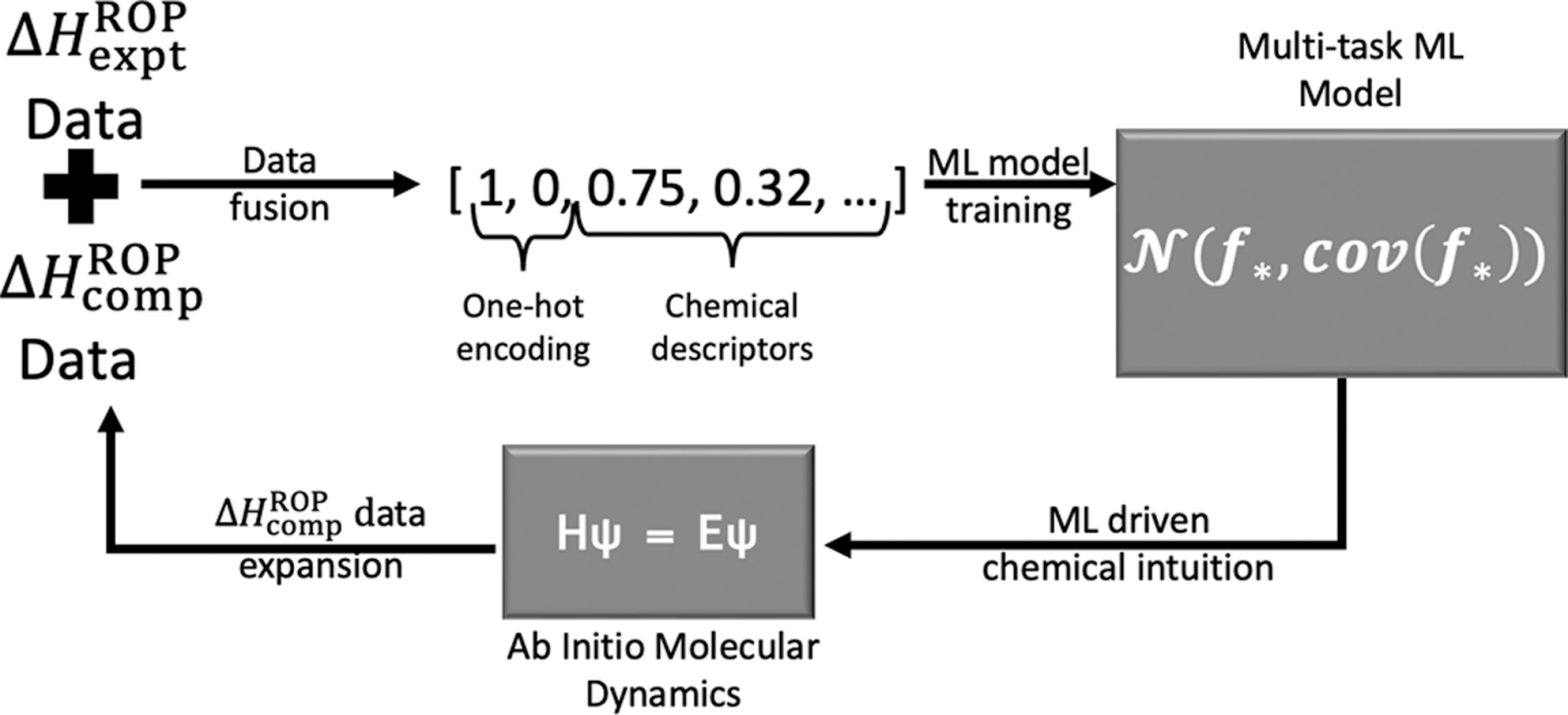
Flowchart describing the overall computational workflow: an initial data set of both Δ*H*_expt_^ROP^ and Δ*H*_comp_^ROP^ is vectorized in such a way that the data source (Δ*H*_expt_^ROP^ or Δ*H*_comp_^ROP^) as well as the chemistry present are machine readable. Next, the multitask model to predict Δ*H*^ROP^ is trained. Then, with this ML model and chemical intuition, the ROP chemical space can be further explored, and the most promising polymers can be suggested to perform additional ab initio computations generating new Δ*H*_comp_^ROP^ data. Then these data can be fed back into training to improve the ML model.

The main objective of this paper is to utilize machine-learning (ML) approaches^26−28^ to build predictive models of Δ*H*^ROP^ trained on data from experiments and the newly developed computational method, i.e., Δ*H*_expt_^ROP^ and Δ*H*_comp_^ROP^. The reason for using two sources of data, experiments and computations, is the following: while experimental data constitute the ground truth, it is typically limited and tends to grow slowly. On the other hand, computational data, although full of built-in approximations owing to practicality, can be produced at scale, grown rapidly, and span new chemical spaces not seen in experimental investigation. As the training data of the model comes from two different sources, a multitask machine-learning approach^29,30^ was utilized. The main motivation of a multitask learning algorithm/model is that by simultaneously learning multiple targets, Δ*H*_expt_^ROP^ and Δ*H*_comp_^ROP^, the underlying correlations between them can be exploited and transferred to the model,^31^ making it more robust and generalizable (to new chemical spaces) than an ML model trained on just the ground-truth experimental data set independently, otherwise known as a single-task model. Such ML approaches have helped design new-to-the-world polymers possessing attractive properties in the past.^2,32,33^ Toward this goal, we have generated and/or curated a comprehensive database of experimentally measured and computed Δ*H*^ROP^, namely, Δ*H*_expt_^ROP^ and Δ*H*_comp_^ROP^, and developed an ML model to instantly predict Δ*H*_expt_^ROP^ for new chemistries. This work focuses particularly on ROP chemistries as this class of polymers has repeatedly shown promise in producing polymers that can be recycled chemically.^10,12,15,17−19^Figure 1 shows the overall pipeline enabled by the newly developed ML model. In the subsequent part of Section 2, we describe all of the critical components of the machine-learning approach to Δ*H*^ROP^, including data generation and capture, polymer fingerprinting, and learning architectures and evaluations.

Going forward, the Δ*H*^ROP^ prediction model will (1) be extended to handle progressively more novel chemistries as newer data become available, (2) inform the next rounds of experiments and computations with attractive Δ*H*^ROP^ and other property values, and ultimately, (3) aid in the accelerated design of depolymerizable and functional polymers.

## 2 Methodology

### 2.1 Experimental Δ*H*_expt_^ROP^ Data Capture

Capturing experimental data from the scientific literature is generally nontrivial, requiring significant time and human effort. Thus, in order to significantly reduce the time required to curate a comprehensive Δ*H*_expt_^ROP^ data set, a natural language processing (NLP) based information extraction (IE) technique to get Δ*H*_expt_^ROP^ data from literature was employed, building on recent work.^34^ Starting from millions of HTML/XML formatted articles, the procedure then occurred in four steps, including (1) document parsing, converting original documents to a format that is suitable for NLP, (2) coarse-grained filtering, where appropriate keywords were used to downselect several to thousands of articles from the initial set, (3) extracting useful information from the downselected papers, and (4) validating the extracted data by domain experts.

In this procedure, step 3 includes three substeps, i.e., (3a) target sentence identification, (3b) material name identification, and (3c) linking material to property. In (3a), heuristic rules were employed to identify candidate sentences. They included searching for sentences containing property names, e.g., enthalpy of polymerization, and units, e.g., kJ/mol or kcal/mol. In (3b), two models were used to identify the compound names. The first model is ChemDataExtractor,^35^ an open-source Python library that extracts chemical names using regular expression (i.e., regex patterns), and the second model is a BERT-based named entity extraction model^36^ trained on a data set of sentences with manually labeled polymer names. Linking the identified material names to property values, which can be formulated as a relation extraction task, was performed in (3c). In this substep, the last material appearing before the property name is regarded as the owner of the property. These methods resulted in an NLP augmented literature search that greatly improved the speed of the data extraction and, as a result, the amount of Δ*H*_expt_^ROP^ data. With the aid of these methods, the Δ*H*_expt_^ROP^ data set was expanded from 88 manually collected data points to 109 data points, resulting in an approximate 24% increase of Δ*H*_expt_^ROP^ data.

### 2.2 Computed Δ*H*_comp_^ROP^ Data Generation

In this work, Δ*H*_comp_^ROP^ was generated using the multistep procedure developed in ref (18). First, a series of closed loops comprised of *L* monomer repeat units were constructed using Polymer Structure Predictor.^37,38^ These loops are representations of polymers. As *L* → ∞, the loop approaches the true polymer limit, and *L* = 1 represents the monomer. The computations were generally performed for *L* = {1, 3, 4, 5, 6}. A classical molecular dynamics (MD) simulation using an empirical Reax force field^39^ was performed for each monomer/polymer model, thoroughly exploring the configuration space while preserving the atomic connectivity. Using classical MD, trajectories of over 1 ns were generated and thousands of snapshots were obtained and sampled to maximize the diversification of the sample set to then be used in *ab initio* MD simulation. The purpose of this step, using classical MD, is to provide a set of maximally diverse initial atomic structures on which to run *ab initio* MD. None of the data generated by classical MD are used to calculate Δ*H*_comp_^ROP^, and thus, no data resulting from classical MD are part of the Δ*H*_comp_^ROP^ data used in subsequent multitask learning. For further information regarding the exact parameters used to run the classical MD simulation to generate initial structures for *ab initio* MD, see Supporting Information.

Next, a room-temperature *ab initio* MD simulation was performed for each sample, obtaining the lowest-energy equilibrated trajectory. The *L*-dependent estimation of Δ*H*_comp_^ROP^ was then computed as , where *E*_*L*_ and *E*_1_ are the potential energies at equilibration of the *ab initio* MD trajectories of the polymer model (*L* > 1) and monomer model (*L* = 1), respectively, while ⟨···⟩ stands for the average over the ensemble of the microstates. Finally, Δ*H*_comp_^ROP^ was defined and computed as the *L* → ∞ (or, equivalently, 1/*L* → 0) limit of Δ*H*_*L*_^ROP^, that is Δ*H*_comp_^ROP^ ≡ lim_*L*→∞_ Δ*H*_*L*_^ROP^. In ref (18), Δ*H*_comp_^ROP^ was computed by assuming that Δ*H*_*L*_^ROP^ depends linearly on 1/*L* and then making suitable extrapolations to the limit of 1/*L* → 0. For the development of our target ML model in this work, Δ*H*_*L*_^ROP^ data will be used directly as training data, i.e., the dependence of Δ*H*_*L*_^ROP^ on *L* will be learned implicitly by the selected ML algorithms. Technical details of this plan can be found in Section 2.4.

The central idea of this computational scheme is that polymers are soft materials; thus, they are naturally not locked at any specific atomic configuration but rather switch across multiple microstates continuously and rapidly. Therefore, this scheme was designed to thoroughly explore the configuration space at two levels: The first is in a “coarse-grained” fashion, using a Reax force field with Large-scale Atomic/Molecular Massively Parallel Simulator (LAMMPS).^40^ The second is using Density Functional Theory (DFT) with Vienna *Ab initio* Simulation Package (vasp).^41,42^ The energies relevant to Δ*H*_comp_^ROP^ are computationally averaged over an ensemble of microstates at the DFT level. While it has been shown that these methods can lead to very accurate predictions for Δ*H*_expt_^ROP^ via linear extrapolation,^18^ it should be noted that the type of long-range polymer dynamics necessary to predict Δ*H*_expt_^ROP^ with certainty cannot fully be accounted for with DFT alone. More details on the computational scheme can be found in ref (18).

### 2.3 Data Summary

**Figure 2:**
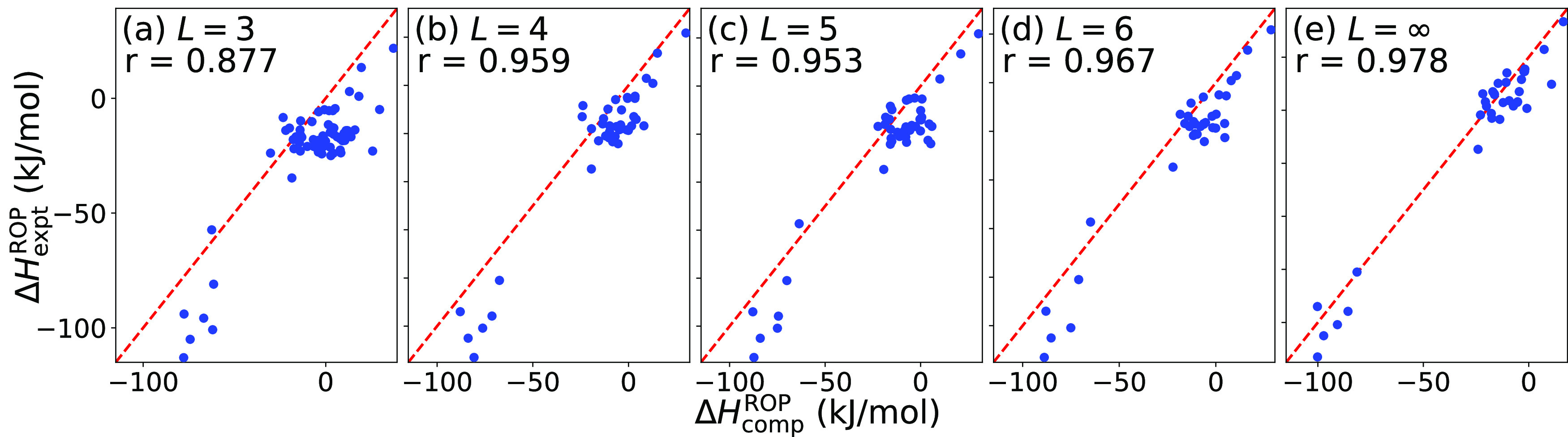
Correlations between Δ*H*_expt_^ROP^ and Δ*H*_L = N_^ROP^, shown for (a) *L* = 3, (b) *L* = 4, (c) *L* = 5, (d) *L* = 6, and (e) *L* = ∞. In the plots, *r* corresponds to the Pearson correlation between Δ*H*_expt_^ROP^ and Δ*H*_comp_^ROP^ and indicates how well-correlated the variables are for a given *L*.

Table 1 provides a summary of our data set, which contains 193 unique ROP polymers and corresponding Δ*H*_expt_^ROP^ and/or Δ*H*_comp_^ROP^. Among them, 109 ROP polymers have been studied experimentally with Δ*H*_expt_^ROP^ values available, while for the remaining 84 polymers, only Δ*H*_comp_^ROP^ data are available. Within the first subset (of 109 ROP polymers for which Δ*H*_expt_^ROP^ data are available), Δ*H*_comp_^ROP^ was computed for 68 polymers, leaving 41 polymers with Δ*H*_expt_^ROP^ only. The “overlap” of 68 polymers that have both Δ*H*_expt_^ROP^ and Δ*H*_comp_^ROP^ is important for our work because, as revealed in Figure 2, experimental data and computed data are strongly correlated (with the correlation increasing with increasing L value). The main objective of multitask learning is to learn and incorporate such correlations implicitly in the ML model targets (Δ*H*_expt_^ROP^ and Δ*H*_comp_^ROP^), making the ML model more robust for cases for which Δ*H*_expt_^ROP^ is not available.

**Table 1:** Summary of the Δ*H*^ROP^ Data Generated, Accumulated, and Used Herein

category	number	Δ*H*_*L*=3_^ROP^	Δ*H*_*L*=4_^ROP^	Δ*H*_*L*=5_^ROP^	Δ*H*_*L*=6_^ROP^
polymers w/Δ*H*_expt_^ROP^ only	41				
polymers w/Δ*H*_comp_^ROP^ only	84	83	26	28	25
polymers w/both Δ*H*_expt_^ROP^ & Δ*H*_comp_^ROP^	68	66	42	45	35
polymers w/either Δ*H*_expt_^ROP^ or Δ*H*_comp_^ROP^	193	149	68	73	60

The subset of Δ*H*_comp_^ROP^ contains 84 + 68 = 152 unique ROP polymers and 428 data points, which can be broken down to 199 data points for Δ*H*_*L* = 3_^ROP^, 78 data points for Δ*H*_*L* = 4_^ROP^, 86 data points for Δ*H*_*L* = 5_^ROP^, and 65 data points for Δ*H*_*L* = 6_^ROP^. Given the nature of our first-principles computational scheme, the generation of Δ*H*_comp_^ROP^ can be performed in a high-throughput, consistent, and targeted manner, i.e., Δ*H*_comp_^ROP^ can be generated for certain polymers so that the training data can be diversified and the target ML model can become progressively more robust with respect to new chemistries.

### 2.4 Polymer Data Fingerprinting

The generated/curated polymer data must be represented (fingerprinted) in machine-readable numerical form before they can be used to train the targeted ML model.^27,28,43^ Our data of Δ*H*^ROP^ contain three classes of information, including the chemical structure of the polymers, usually given in terms of a SMILES string,^28,44^ the nature of Δ*H*^ROP^, i.e., whether the data point is from experimental or computed sources (specified as (1, 0) or (0, 1), respectively), and the loop size specified as  (with  for all Δ*H*_expt_^ROP^ data). Using the hierarchical fingerprinting procedure that was developed^27,28,43^ during the past decade and currently used in Polymer Genome^27,28^ the polymer SMILES is converted into a numerical vector of over 200 dimensions (or columns) to represent the chemical structure of the polymers. The three classes of information (chemical, data source, and ) were stacked into a composite fingerprint that was then mapped onto the target properties, i.e., Δ*H*_expt_^ROP^ and Δ*H*_comp_^ROP^. Feature engineering, namely, permutation feature engineering, was subsequently used for each machine-learning algorithm tested in Section 3.1 to reduce the number of dimensions of the overall fingerprint to 80. This procedure is generic and can be used to prepare training data emerging from multiple sources. Consequently, it has been widely used for multitask learning efforts within the area of Materials Informatics.^31,32,45−47^ With a scheme for creating the training data fingerprints for multitask ML, a suitable algorithm is needed to map the composite fingerprints onto the targeted property values. Four algorithms that are suitable for small training data sets, including Support Vector Machine (SVM), Random Forest (RF), Boosted Random Forest (BRF), and Gaussian Process Regression (GPR), were tested to determine the best learning technique for our data. The results for each learning algorithm are described in the following sections.

## 3 Results and Discussion

### 3.1 Machine-Learning Models and Validation

The four algorithms considered were evaluated in a customized leave-one-out cross-validation (LOOCV) protocol in which a held-out polymer, for which Δ*H*_expt_^ROP^ is available, is targeted and predicted by the ML models trained with four different training set schemes (also referred to as “cases”). These cases were designed to systematically examine and reveal the role of Δ*H*_comp_^ROP^, the subsequent benefit of multitask learning, and the performance of the developed models. These four cases are summarized in Table 2. In the first case, only the available experimental data were used for training, so the model is “effectively” a single-task (ST) model, and so, this case is named ST. The next three cases are MT1, MT2, and MT3, which are designed to gradually supply the (multitask) learning algorithms with selected subsets of computational data, i.e., Δ*H*_comp_^ROP^, and, consequently, gradually improve ML models. Among three multitask (MT) cases, MT1 does not include computed data of any size (L) for the held-out polymer. This simulates the case when there is no computational data available for the polymer of interest being predicted. The MT2 case assumes that there is minimal computational data available, i.e., just corresponding to *L* = 3, in the training data for the held-out polymer. Finally, the MT3 case represents the situation where plenty of computational data are available for the held-out polymer being predicted.

**Table 2:** Summary of Four Cases Used in Evaluating the ML Algorithms, Which Are Different in the Training Data

case	training data
ST	experimental data only
MT1	experimental data + computed data, excluding all Δ*H*_comp_^ROP^ computed for the held-out polymer
MT2	experimental data + computed data in which only Δ*H*_*L* = 3_^ROP^ computed for the held-out polymer is included
MT3	experimental data + all computed data, including Δ*H*_*L* = *N*_^ROP^ for all N computed for the held-out polymer

Table 3 shows two error metrics, i.e., the root-mean-square error (RMSE) and the determination coefficient (*R*^2^) obtained by using SVM, RF, BRF, and GPR for all 4 cases, namely ST, MT1, MT2, and MT3. The presented results were obtained by (1) selecting a held-out polymer for which Δ*H*_expt_^ROP^ is available, (2) preparing the training data for the four cases as defined in Table 2, (3) using a learning algorithm to train an ML model for each training data set, (4) making predictions on the held-out polymer, and (5) screening over all the possible (68) held-out polymers to get the prediction metrics (i.e., RMSE and *R*^2^). Hyper-parameters for a given ML algorithm were chosen using 5-fold cross-validation and a grid approach, where all permutations of a list of hyper-parameters were tested prior to the LOOCV scheme described above. In step (5), for the sake of a fair comparison, the held-out polymer was selected in the subset of 68 unique polymers for which both Δ*H*_expt_^ROP^ and Δ*H*_comp_^ROP^ are available.

**Table 3:** RMSE, Given in kJ/mol, and *R*^2^ Obtained from SVM, RF, BRF, and GPR for Different Cases Described in the Text

	ST		MT1		MT2		MT3	
model type	RMSE	*R*^2^	RMSE	*R*^2^	RMSE	*R*^2^	RMSE	*R*^2^
RF	8.3	0.89	10.7	0.87	10.0	0.85	8.8	0.88
SVM	17.1	0.55	11.2	0.81	10.5	0.83	9.2	0.88
BRF	9.3	0.87	9.4	0.87	9.7	0.86	9.0	0.88
GPR	12.2	0.77	9.2	0.87	8.8	0.88	8.0	0.90

**Figure 3:**
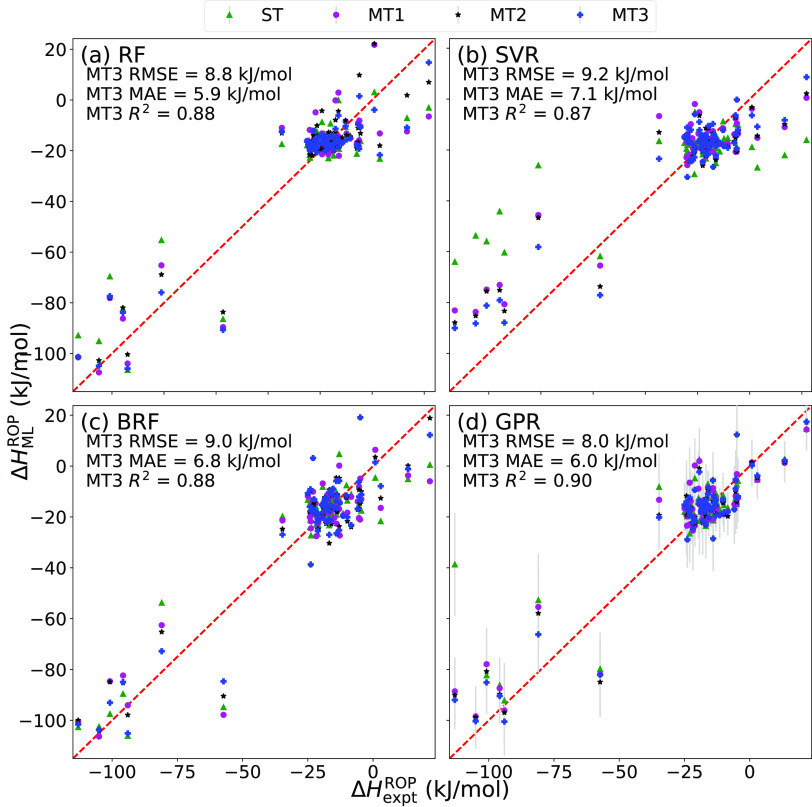
Predicted Δ*H*_expt_^ROP^, given in a comparison with the ground truth, i.e., the actual values of Δ*H*_expt_^ROP^, of 68 polymers for which both Δ*H*_expt_^ROP^ and Δ*H*_compt_^ROP^ are available. Results obtained from cases ST, MT1, MT2, and MT3 are shown in (a)–(d), respectively.

The obtained results, which are shown in Table 3, demonstrate that by combining computed data and experimental data, the trained (multitask) ML models are improved in accuracy. In terms of RMSE and *R*^2^, the best algorithm to learn our Δ*H*_expt_^ROP^ data is GPR, as has widely been shown in the literature for small data sets, especially polymer data.^27,28,46,48−51^ Using GPR, RMSE is reduced from 12.2 kJ/mol for ST (trained only on experimental data) to 9.2 kJ/mol for MT1, 8.8 kJ/mol for MT2 and 8.0 kJ/mol for MT3. This MT3 value comes close to the desired chemical accuracy, which is about 5 kJ/mol. Therefore, GPR^52^ was selected for the eventual development of the predictive ML “production” model of Δ*H*_expt_^ROP^. Figure 3 visualizes the predictions performed for all the possible (68) held-out polymers in all four cases, given with respect to the ground truth, i.e., Δ*H*_expt_^ROP^ for each of the four algorithms tested (RF, SVM, BRF, and GPR).

Some valuable notes can be drawn from the LOOCV analysis. First, the performance of ST models for all algorithms does not show satisfactory enough accuracy. We attribute this to be due to data scarcity, and it is the motivation for why such a large Δ*H*_comp_^ROP^ data set was developed and multitask learning was employed. Next, in the case of GPR, adding in computed data that are not associated with the held-out polymer (MT1) improves the model’s accuracy for predicting the unseen polymer. This is seen in the improvement of both RMSE and *R*^2^ seen from ST to MT1. We believe this improvement from ST to MT1 is due to greater generalizability of the model as a result of greater chemical coverage represented in the computational data and thus evidence of the benefit of multitask learning. Second, significant improvement is seen from MT1 to MT2 where only the computationally least expensive *ab initio* MD computation is performed. This suggests that computed Δ*H*_comp_^ROP^, especially Δ*H*_*L* = 3_^ROP^, can be done in a high-throughput manner, in order to develop a multitask model that can predict Δ*H*_expt_^ROP^ for the cases of interest with satisfactory accuracy. Lastly, for all algorithms except for RF we see yet another improvement on going from MT2 to MT3, which shows that additional Δ*H*_comp_^ROP^ of various sizes helps the ML models improve their ability to extrapolate to the experimental case.

### 3.2 Production Model

Given the analysis described in Section 3.1, we concluded that GPR is the algorithm of choice to develop a production multitask ML model that is trained on all Δ*H*_comp_^ROP^ and Δ*H*_expt_^ROP^ data. The main objective of this model is to predict the Δ*H*_expt_^ROP^ from the chemical structure, or the SMILES, of the polymer that is obtained by opening a ring monomer. Because GPR returns not only the target value prediction but also an intrinsic measure of the prediction uncertainty,^52^ the selection of GPR for the production model has an extra advantage. Given a new polymer, a large prediction uncertainty clearly indicates that the chemistry of the polymer is not very well represented in the training data, and in this case, performing some computations for Δ*H*_comp_^ROP^, especially Δ*H*_*L* = 3_^ROP^, can not only significantly improve the prediction but also improve the production model in general.

**Figure 4:**
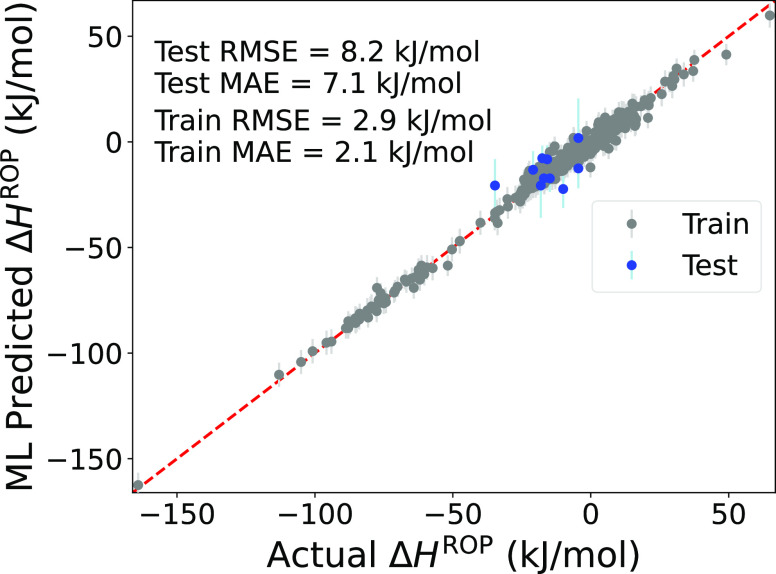
Parity plot for the preproduction model where 10% of the Δ*H*_expt_^ROP^ data were withheld. Blue data points represent the test data, while gray data points represent the train data (which contain both Δ*H*_comp_^ROP^ and Δ*H*_expt_^ROP^).

To assess potential overfitting, a preproduction model was considered in which 10 Δ*H*_expt_^ROP^ data points (10% of the experimental data set) were randomly withheld from training, such that 5 of the data points had Δ*H*_comp_^ROP^ in the training set and 5 data points did not have Δ*H*_comp_^ROP^ available. The obtained model had a training RMSE of 2.9 and a test RMSE of 8.2 kJ/mol. These results are visualized in Figure 4, which includes Δ*H*_comp_^ROP^ and Δ*H*_expt_^ROP^ data. Further the test mean absolute error (MAE) is in line with 7 kJ/mol, which is significant as this is the approximate accuracy reported when linearly extrapolating from multiple Δ*H*_comp_^ROP^ of different sizes to the case of an infinite-sized model.^18^

**Figure 5:**
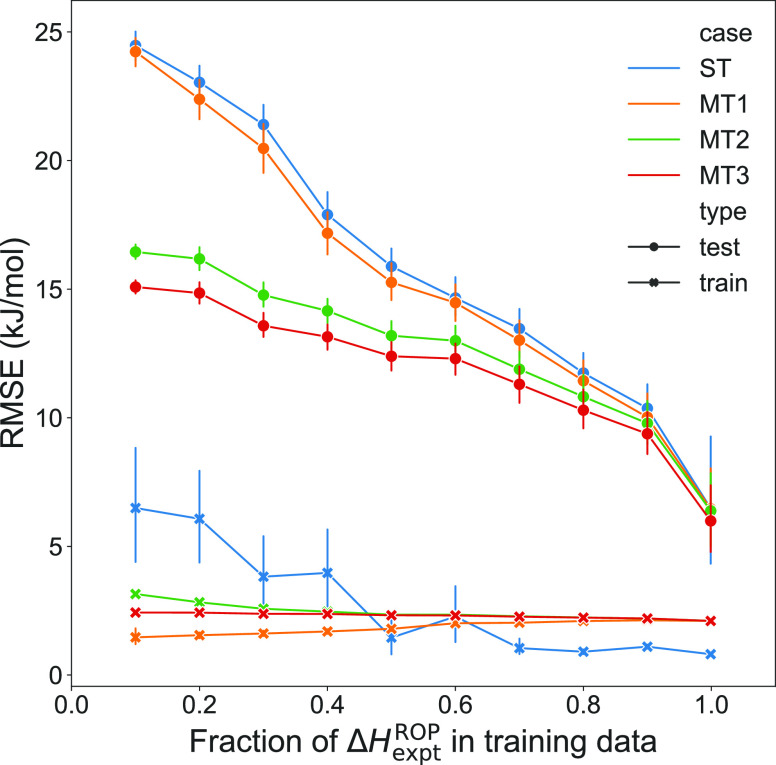
Learning curve for the different cases as described in the Table 2. Here, each case is indicated by a different color. The shape of the marker indicates between test and train performance, where the x indicates train and the dots indicate test.

For all cases of testing the model, it seems due to data scarcity that data performance is limited. This can be seen as a large difference between test and train RMSEs and in the fact that there is no leveling of the test curves for any case of Δ*H*_comp_^ROP^ data availability in Figure 5. To generate Figure 5, test train splits that varied from 10% train and 90% test to 99% train and 1% test were randomly split among the Δ*H*_expt_^ROP^ data. In each of these splits, the split was done randomly and 100 times in order to collect statistics for how different random splits could affect the accuracy of the trained ML model, allowing for the error bars to be plotted. In this random splitting of Δ*H*_expt_^ROP^, the Δ*H*_comp_^ROP^ data subsequently added to the training was intentionally modified so the same cases outlined in Table 2 were tested in the learning curve as well. Figure 5 shows the importance of continued data expansion, and while experimental data are the highest fidelity data that can be used for model training and evaluation, the expansion of DFT data is much easier and faster to perform. Further, from the results of the LOOCV analysis specifically for the GPR algorithm shown in Figure 3 and Table 3, it seems evident that loop size 3 DFT data, i.e., Δ*H*_*L* = 3_^ROP^, the cheapest data to gather from a time and computational resource standpoint, are significantly helpful in obtaining better predictions. Thus, in an effort to continue to improve the models, the ROP chemical space will continue to be searched first with Δ*H*_*L* = 3_^ROP^ computations in an effort to best create an ML model that can generalize to diverse chemistries.

Finally, the production model was developed using GPR and the choice of kernel was discovered to be optimal during LOOCV. Just prior to this, a 10-fold cross-validation was performed to achieve an average train RMSE of 1.55 kJ/mol and an average test RMSE of 8.80 kJ/mol. It is evident that overfitting is still present, but this is a common problem with a training data size of a few hundred as we have in this work. This work will continue and the production model will constantly be updated by training on new Δ*H*_comp_^ROP^ data that is generated.

## 4 Conclusions

In this work, we have developed a largest-of-its-kind data set of Δ*H*^ROP^, which consists of data from both experimental measurements and high-throughput computations using a recently developed first-principles scheme.^18^ This data set was then leveraged to develop a multitask ML model that can predict the experimental value of Δ*H*^ROP^ with an accuracy of 8 kJ/mol that approaches the (gold standard) chemical accuracy of about ≃5 kJ/mol. Given its high accuracy, this model is expected to contribute to the development of depolymerizable polymers via ROP. Polymers synthesized via ROP are focused on particularly in this work due to their shown potential in literature to create polymers that have the necessary polymerization thermodynamics to be depolymerized. Data from future experiments and computations will be used to further improve this model.

## Supporting Information Available

In addition, all experimental and computational data are also provided in excel format along with chemical structures and SMILES strings in two different formats (“long” and “wide”). All codes and the data files may also be found in the Ramprasad github at https://github.com/Ramprasad-Group/enthalpy_ml_paper_code (ZIP)
A table in PDF format detailing the experimental data collected from the literature is provided as part of the SI (PDF)
