# Supplementary material for: Accelerated Scheme to Predict Ring-Opening Polymerization Enthalpy: Simulation-Experimental Data Fusion and Multitask Machine Learning
Source: J Phys Chem A. 2023 Dec 6;127(50):10709–16. doi: 10.1021/acs.jpca.3c05870 (PMC10749451; doi:10.1021/acs.jpca.3c05870)
Supplement: Supplementary file 2 — jp3c05870_si_002.pdf [file jp3c05870_si_002.pdf]

Supporting Information

Accelerated Scheme to Predict Ring-Opening Polymerization Enthalpy: Simulation-Experimental Data Fusion and Multi-Task Machine Learning

Aubrey Toland[1], Huan Tran[1], Lihua Chen[1], Yinghao Li[2], Chao Zhang[2], Will Gutekunst[3], and Rampi Ramprasad*[1]

[1]*School of Materials Science & Engineering, Georgia Institute of Technology, Atlanta, Georgia, 30332, USA*
[2]*School of Computational Science and Engineering, Georgia Institute of Technology, Atlanta, Georgia, 30332, USA*
[3]*School of Chemistry and Biochemistry, Georgia Institute of Technology, Atlanta, Georgia, 30332, USA*

E-mail: rampi.ramprasad@mse.gatech.edu*

1 Data Availability

We disclose the entire experimental dataset collected from literature in pdf form below in Section 2. Additional files containing all data, both experimental and ab initio MD data, are available as supplementary files. These supplimentary files are ROP-enthalpy-data-long.csv and ROP-enthalpy-data-wide.xlsx, and both represent the same data in separate ways. The columns of both are listed and described below:

- ROP-enthalpy-data-long.csv

    1. **ID**: an integer identifier for a given polymer
    2. **smiles_polymer**: The smiles for the polymer
    3. **smiles_monomer**: The smiles for the monomer
    4. **1/length**: The inverse of the number of repeat units used in the calculation, rounded to three places. For experimental data this value is listed as 0
    5. **source**: indicate whether the data is from experimental measurement or DFT calculations

6. **roe_kj/mol**: The enthalpy value associated with the datapoint

- ROP-enthalpy-data-wide.xlsx

1. **ID**: an integer identifier for a given polymer
2. **smiles_polymer**: The smiles for the polymer
3. **smiles_monomer**: The smiles for the monomer
4. **monomer_image**: an image of the monomer
5. **delta_H_exp_KJ/mol**: $\Delta H^{\mathrm{ROP}}_{\mathrm{expt}}$ in kJ/mol
6. **delta_H_1/l-3_KJ/mol**: $\Delta H^{\mathrm{ROP}}_{\mathrm{L=3}}$ in kJ/mol
7. **delta_H_1/l-4_KJ/mol**: $\Delta H^{\mathrm{ROP}}_{\mathrm{L=4}}$ in kJ/mol
8. **delta_H_1/l-5_KJ/mol**: $\Delta H^{\mathrm{ROP}}_{\mathrm{L=5}}$ in kJ/mol
9. **delta_H_1/l-6_KJ/mol**: $\Delta H^{\mathrm{ROP}}_{\mathrm{L=6}}$ in kJ/mol
10. **reference**: DOI for the paper that the data point comes from if there is experimental data

2 Experimental table

Monomer Image	$\Delta H^{\mathrm{ROP}}_{\mathrm{exp}}$ (kJ/mol)	Reference (doi)
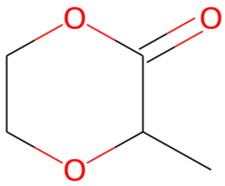	−9.85	10.1002/pi.2871
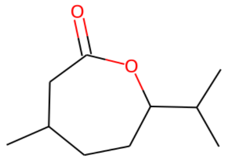	−16.8	10.1021/bm050076t
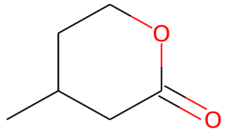	−13.8	10.1073/pnas.1404596111

Monomer Image	$\Delta H^{\mathrm{ROP}}_{\mathrm{comp}}$ (kJ/mol)	Reference (doi)
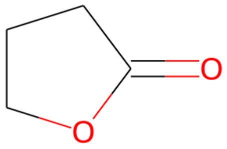	−5.4	10.1038/nchem.2391
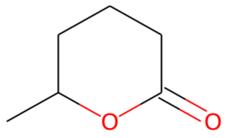	−13.8	10.1002/1521-3935(20020401)203:5/6<889::AID-MACP889>3.0.CO;2-O Phys. 2002, 203, 889
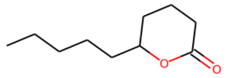	−17.1	10.1021/mz200006s
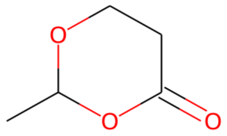	−11.6	10.1021/mz5005794
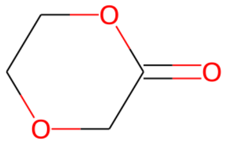	−14.1	10.1021/ma000457t
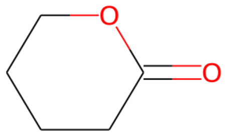	−8.4	10.1002/1521-3935(20020401)203:5/6<889::AID-MACP889>3.0.CO;2-O
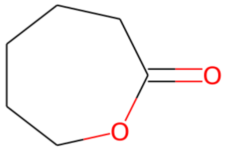	−14.0	10.1002/1521-3935(20020401)203:5/6<889::AID-MACP889>3.0.CO;2-O

Monomer Image	$\Delta H^{\mathrm{ROP}}_{\mathrm{comp}}$ (kJ/mol)	Reference (doi)
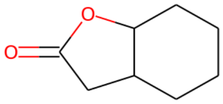	−18.0	10.1038/pj.2013.70, 10.1126/science.aar5498
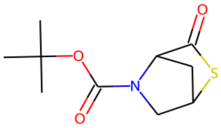	−15.6	10.1021/jacs.9b00031
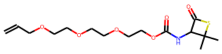	−9.4	10.1016/j.chempr.2020.06.003
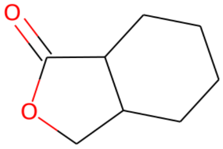	−20.0	10.1126/science.aar5498
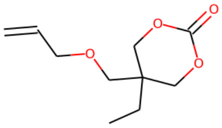	−22.0	10.1021/acs.biomac.6b01375
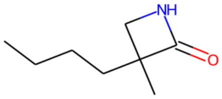	−79.84	10.1002/pol.1976.170141003
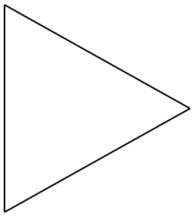	−113.0	10.1039/QR9581200061

Monomer Image	ΔH_{comp}^{ROP} (kJ/mol)	Reference (doi)
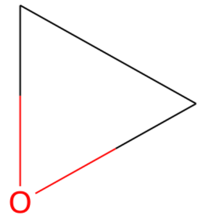	−94.0	10.1246/bcsj.20170073
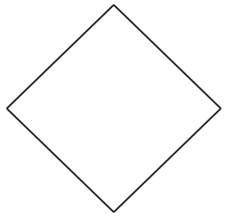	−105.01	10.1039/QR9581200061
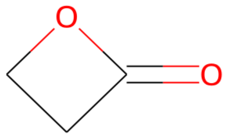	−81.0	Thermodynamics and kinetics of ring-opening polymerization by Andrzej Duda and Adam Kowalski, Acta. Chem. Scand. vol. 30, no. 3, p. 14 (1966)
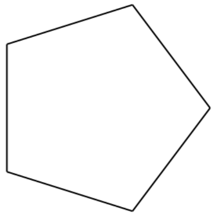	−21.2	10.1039/QR9581200061
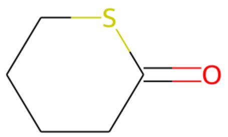	−4.9	10.1021/acsmacrolett.2c00319
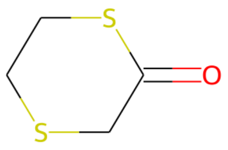	−9.5	10.1021/acsmacrolett.2c00319
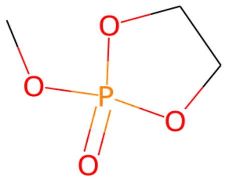	−14.0	10.1002/marc.1984.030050501

Monomer Image	$\Delta H^{\mathrm{ROP}}_{\mathrm{comp}}$ (kJ/mol)	Reference (doi)
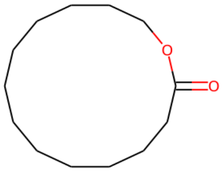	−8.0	10.1002/pol.1966.150041208
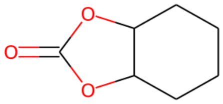	−23.0	10.1038/pj.2013.50
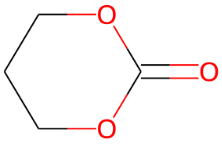	−26.0	10.1021/ma971227q
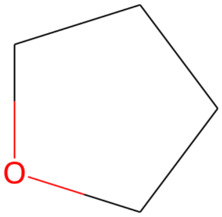	−24.0	10.1016/0032-3861(89)90162-6
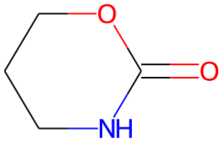	−18.0	10.1002/macp.1997.021980104
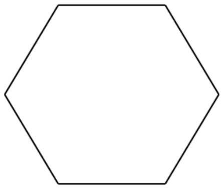	2.9288	10.1039/TF9555101710, 10.1039/QR9581200061
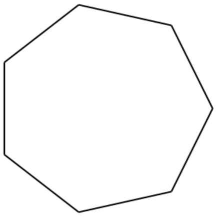	−21.7568	10.1039/TF9555101710

Monomer Image	$\Delta H^{\mathrm{ROP}}_{\mathrm{comp}}$ (kJ/mol)	Reference (doi)
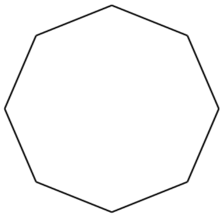	−34.7272	10.1039/TF9555101710
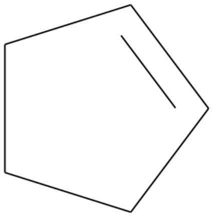	−23.43	10.1039/c3py00584d
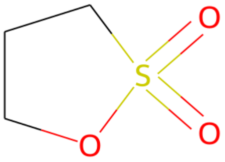	−23.012	10.1295/polymj.6.238
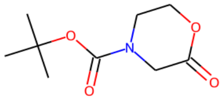	−19.63	10.1021/ja503830c
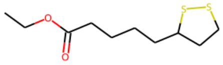	−15.7	10.1002/pol.20200765
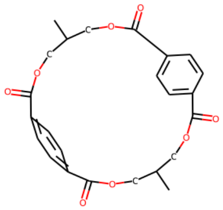	−1.07	10.1007/s10118-018-2161-4
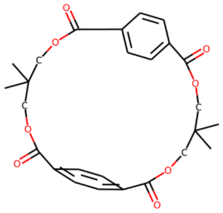	−2.67	10.1007/s10118-018-2161-4

Monomer Image	$\Delta H^{\mathrm{ROP}}_{\mathrm{comp}}$ (kJ/mol)	Reference (doi)
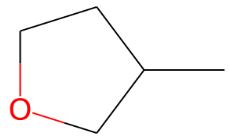	−22.59	10.1021/ma50005a048
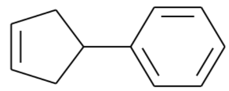	−21.34	10.1002/marc.201600121
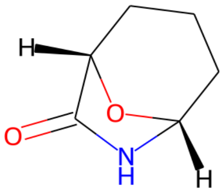	−23.8	10.1002/pol.1989.140270905
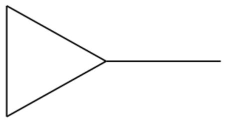	−100.83	10.1039/TF9555101710
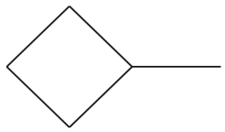	−95.81	10.1039/TF9555101710
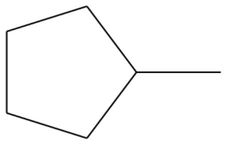	−12.97	10.1039/TF9555101710
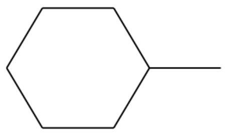	13.39	10.1039/TF9555101710

Monomer Image	$\Delta H^{\mathrm{ROP}}_{\mathrm{comp}}$ (kJ/mol)	Reference (doi)
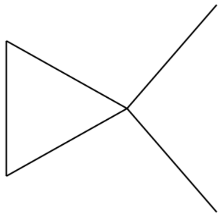	−57.32	10.1039/TF9555101710
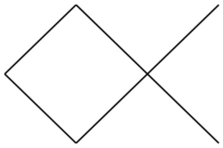	−85.35	10.1039/TF9555101710
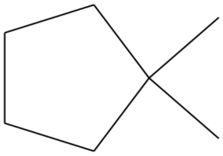	0.84	10.1039/TF9555101710
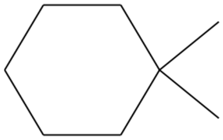	21.76	10.1039/TF9555101710
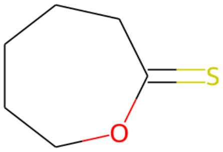	−24.23	10.1039/D0PY01393E
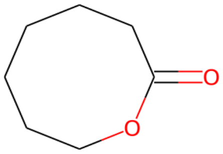	−19.25	10.1039/D0PY01393E
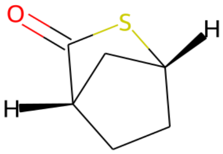	−14.1	10.1126/sciadv.abc0495

Monomer Image	$\Delta H^{\mathrm{ROP}}_{\mathrm{comp}}$ (kJ/mol)	Reference (doi)
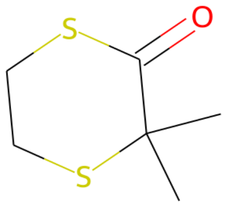	−19.3	10.1021/acsmacrolett.2c00319
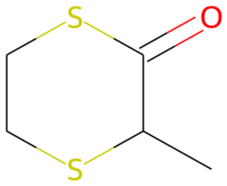	−10.2	10.1021/acsmacrolett.2c00319
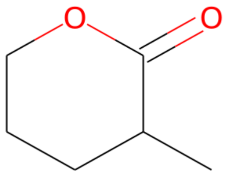	−13.0	10.1021/acs.macromol.6b00211
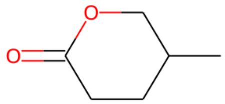	−15.8	10.1021/acs.macromol.6b00211
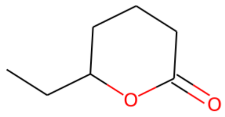	−16.4	10.1021/acs.macromol.6b00211
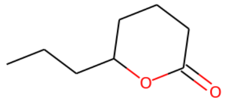	−18.5	10.1021/acs.macromol.6b00211
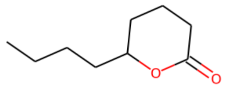	−17.0	10.1021/acs.macromol.6b00211

Monomer Image	$\Delta H_{\text{comp}}^{\text{ROP}}$ (kJ/mol)	Reference (doi)
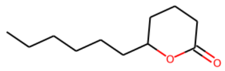	−18.4	10.1021/acs.macromol.6b00211
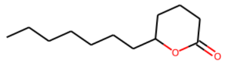	−18.7	10.1021/acs.macromol.6b00211
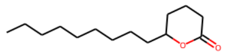	−16.8	10.1021/acs.macromol.6b00211
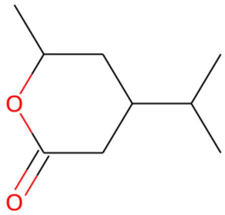	−16.8	10.1021/bm050076t
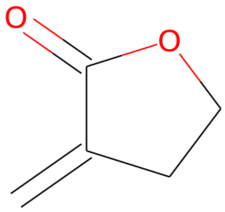	−5.9	10.1021/jacs.6b07974
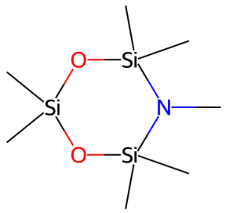	−4.5	10.1021/ma061708m
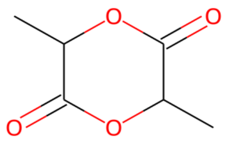	−23.9	10.1021/ma0007177

Monomer Image	$\Delta H^{\mathrm{ROP}}_{\mathrm{comp}}$ (kJ/mol)	Reference (doi)
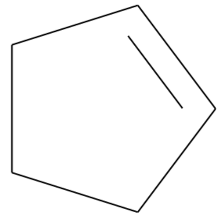	−16.8252222	10.1021/acsmacrolett.8b00422
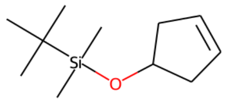	−17.5751901	10.1021/acsmacrolett.8b00422
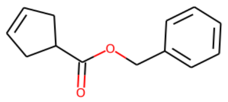	−21.0207189	10.1021/acsmacrolett.8b00422
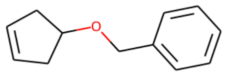	−15.2803881	10.1021/acsmacrolett.8b00422
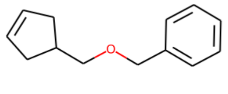	−17.5984707	10.1021/acsmacrolett.8b00422
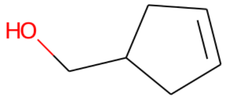	−18.3999885	10.1021/acsmacrolett.8b00422
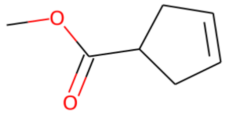	−14.83722525	10.1021/acsmacrolett.8b00422

Monomer Image	$\Delta H_{\text{comp}}^{\text{ROP}}$ (kJ/mol)	Reference (doi)
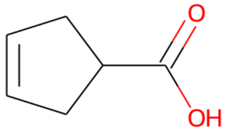	−12.7810494	10.1021/acsmacrolett.8b00422
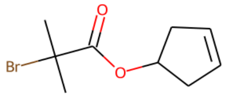	−22.6	10.1021/acsmacrolett.8b00576
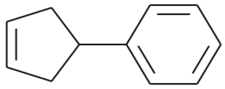	−21.358666	10.1002/marc.201600121
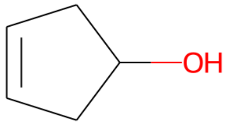	−11.5015876	10.1021/acsmacrolett.8b00422
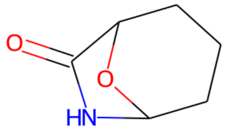	−23.8	10.1016/s0079-6700(00)00018-6
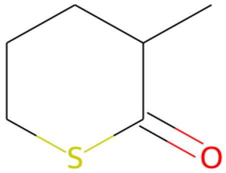	−4.5	10.1021/acsmacrolett.2c00319
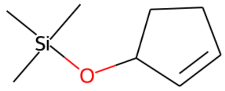	−12.7	10.1016/j.eurpolymj.2019.109251

Monomer Image	$\Delta H^{\mathrm{ROP}}_{\mathrm{comp}}$ (kJ/mol)	Reference (doi)
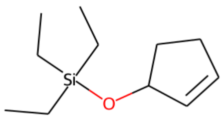	−21.3	10.1016/j.eurpolymj.2019.109251
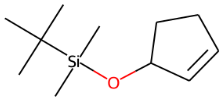	−6.8	10.1016/j.eurpolymj.2019.109251
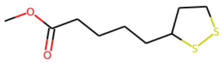	−17.9	10.1021/jacs.9b08957
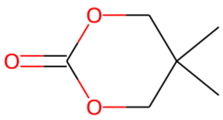	−21.0	10.1021/ma971227q
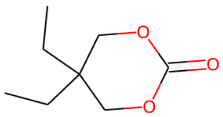	−18.0	10.1021/ma971227q
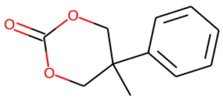	−16.0	10.1021/ma971227q
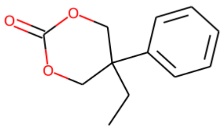	−5.0	10.1021/ma971227q

Monomer Image	$\Delta H_{\mathrm{comp}}^{\mathrm{ROP}}$ (kJ/mol)	Reference (doi)
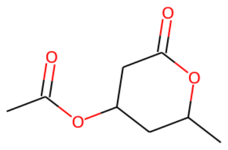	−25.0	10.1039/d1py00561h
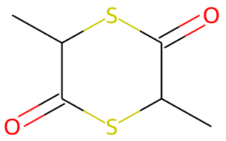	−4.92	10.1002/anie.202109767
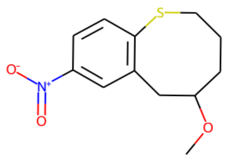	−17.88	doi.org/10.1021/jacs.3c03455
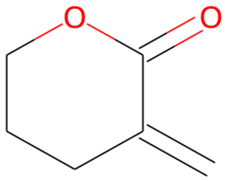	−16.5	10.1021/jacs.6b07974
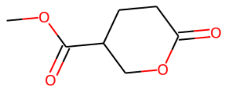	−15.2	10.1021/acsmacrolett.7b00889
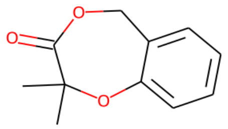	−27.3	10.1021/jacs.1c10162
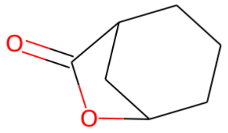	−21.1	10.1016/j.chempr.2021.02.003

Monomer Image	$\Delta H^{\mathrm{ROP}}_{\mathrm{comp}}$ (kJ/mol)	Reference (doi)
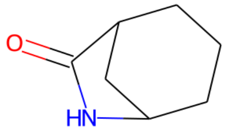	−10.0	10.1021/jacs.1c12611
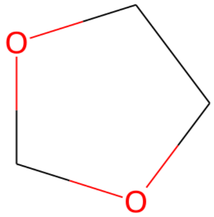	−20.0	10.1126/science.abh0626
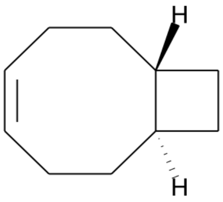	−8.8	10.1021/jacs.1c11197
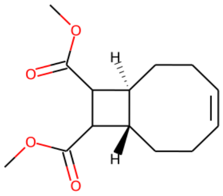	−7.1	10.1038/s41557-021-00748-5
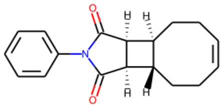	−11.7	10.1038/s41557-021-00748-5
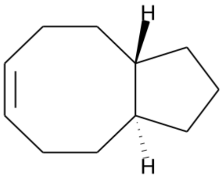	−12.1	10.1021/jacs.1c11197
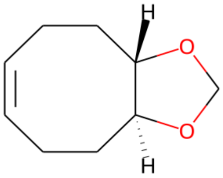	−11.3	10.1021/jacs.1c11197

Monomer Image	$\Delta H^{\mathrm{ROP}}_{\mathrm{comp}}$ (kJ/mol)	Reference (doi)
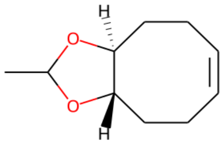	−10.9	10.1021/jacs.1c11197
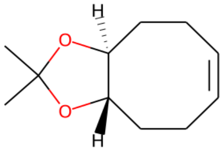	−13.4	10.1021/jacs.1c11197
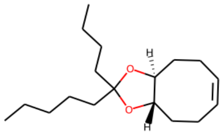	−13.8	10.1021/jacs.1c11197
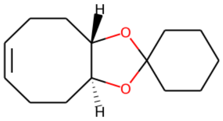	−11.7	10.1021/jacs.1c11197
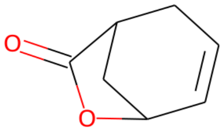	−34.7	10.1021/jacs.1c12278
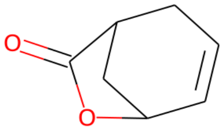	−19.8	10.1021/jacs.1c12278
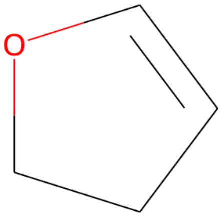	−20.9	10.1021/jacs.9b11834

Monomer Image	ΔH_{comp}^{ROP} (kJ/mol)	Reference (doi)
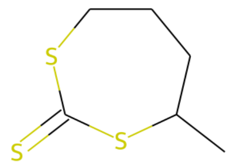	−18.11	10.1021/acs.macromol.2c01628

3 Classical MD Parameters

Classical MD was run using LAMMPS. Classical MD simulations were run to create a pool of initial 3D configurations for then running subsequent *ab-initio* MD powered by DFT. The classical MD simulations were run using the ffield_CHOSFClN force field, a reaxff force field. Further the simulation itself consisted of one NVT held at 300 K for over 1 ns.
